# Exploration of KIR genes and hematological-related diseases in Chinese Han population

**DOI:** 10.1038/s41598-023-36882-y

**Published:** 2023-06-16

**Authors:** Ye-Mo Li, Yu-Xia Li, Xiao-Zhuang Hu, Dai-Yang Li, Lin An, Zhi-Yang Yuan, Zhong-Liang Liu, Ke-Ming Du, Zhong-Zheng Zheng

**Affiliations:** Shanghai Tissuebank Biotechnology Co, Ltd, Shanghai, China

**Keywords:** Genetics, Immunology, Biomarkers

## Abstract

The function of natural killer (NK) cells has previously been implicated in hematopoietic-related diseases. Killer immunoglobulin-like receptors (KIR) play an important role in NK cells after hematopoietic stem cell transplantation. To explore the immunogenetic predisposition of hematological-related diseases, herein, a multi-center retrospective study in China was conducted, analyzing and comparing 2519 patients with hematopathy (mainly, acute lymphoblastic leukemia, acute myeloid leukemia, aplastic anemia, and myelodysplastic syndrome) to 18,108 individuals without known pathology. Genotyping was performed by polymerase chain reaction with specific sequence primers (PCR-SSP). As a result, we discovered four genes including KIR2DL5 (OR: 0.74, 95% CI 0.59–0.93; Pc = 0.0405), 2DS1 (OR: 0.74, 95% CI 0.59–0.93; Pc = 0.0405), 2DS3 (OR: 0.58, 95% CI 0.41–0.81; Pc = 0.0180), and 3DS1 (OR: 0.74, 95% CI 0.58–0.94; Pc = 0.0405) to be protective factors that significantly reduce the risk of aplastic anemia. Our findings offer new approaches to immunotherapy for hematological-related diseases. As these therapies mature, they are promising to be used alone or in combination with current treatments to help to make blood disorders a manageable disease.

## Introduction

As one of the conditions that severely threaten people’s health, hematopoietic-related diseases have gotten a rising tendency in China recently^1^. With the development of molecular immunology and related medical fundamental research, relationships between some susceptibility genes, immune-response genes and natural killer (NK) cell dysfunction and blood disorders have attracted increasing attention. Notably, certain hematopoietic stem cell (HSC) disorders, including aplastic anemia (AA) and myelodysplastic syndrome (MDS), share similar pathogenic pathways^2–5^. Despite breakthroughs in the understanding of immune pathophysiology, numerous undecided issues and conflicting findings still remain. This uncertainty highlights the importance of predicting disease susceptibility to discover immunotherapies that prevent the progression of hematopoietic-related diseases.

Killer cell immunoglobulin-like receptors (KIRs) can inhibit or activate NK cells by recognizing and binding human leukocyte antigen (HLA) class I ligands^6,7^. The KIR gene family consists of 16 genes located on chromosome 19q13.4. There are two pseudogenes (2DP1 and 3DP1), seven inhibitory genes (2DL1, 2DL2, 2DL3, 2DL5, and 3DL1-3DL3), six activating genes (2DS1-2DS5 and 3DS1), along with 2DL4 (activating and inhibiting potential). Several studies revealed that the diversity of KIR genes was related to the number of genes and genotypic variability. Ultimately, it can lead to an understanding of KIR polymorphism if well studied^8,9^.

A rising number of epidemiological studies have demonstrated that the interactions between KIRs and HLA class I ligands are an innate pattern of the immune system, autoimmune diseases, and cancer progression^10–15^. Additionally, KIR genes exhibit genetic susceptibility to a variety of disorders, for instance, KIR2DS2 for rheumatoid arthritis, scleroderma, and type I diabetes^16^. However, the associations between KIR gene polymorphisms and hematopoietic-related diseases susceptibility were limited by small sample size cohort studies. Therefore, it impeded the evaluation and verification of susceptibility genes for diseases^17,18^.

In this study, the hypothesis that “similar immunogenetic predisposition exists in all or specific subgroups of hematology” may be tested. We observed the gene frequency and distribution of KIR genotypes in patients with acute lymphoblastic leukemia (ALL), acute myeloid leukemia (AML), AA, and MDS. Moreover, the relationship between KIR genotypes and these disorders was investigated.

## Results

### Characteristics of KIR genes frequency

A total of 20,627 samples were analyzed from 2519 patients and 18,108 individuals without known pathology. The frequencies of each KIR gene of patients and controls are listed in Table 1. Notably, there were significant differences in 3DL2 (99.64% vs. 99.92%; *P* = 0.0133), 3DL3 (99.64% vs. 99.92%; *P* = 0.0133), and 3DP1 (99.6% vs. 99.87%; *P* = 0.044). The odds ratios (ORs) of 3DL2, 3DL3, and 3DP1 were 0.22 (95% confidence interval [CI]: 0.09–0.50), 0.23 (95%CI: 0.10–0.53), and 0.33 (95%CI: 0.16–0.70), respectively. The difference in other KIR genes was not significant.

**Table 1:** Characteristics of KIR gene frequency in the study population.

	Patients n (%)	Controls n (%)	*P*	OR (95% CI)
*2DL1*	2498 (99.17)	17,965 (99.21)	0.96	0.9469 (0.5978, 1.4999)
*2DL2*	557 (22.11)	3885 (21.45)	0.8236	1.0393 (0.9399, 1.1493)
*2DL3*	2484 (98.61)	17,847 (98.56)	0.96	1.0379 (0.7275, 1.4807)
*2DL4*	2507 (99.52)	18,084 (99.87)	0.0133	0.2773 (0.1385, 0.5552)
*2DL5*	1021 (40.53)	7423 (40.99)	0.96	0.9811 (0.9013, 1.0680)
*3DL1*	2405 (95.47)	17,296 (95.52)	0.96	0.9904 (0.8104, 1.2104)
*3DL2*	2510 (99.64)	18,094 (99.92)	0.0133	0.2158 (0.0933, 0.4991)
*3DL3*	2510 (99.64)	18,093 (99.92)	0.0133	0.2312 (0.1011, 0.5289)
*2DS1*	918 (36.44)	6757 (37.31)	0.8236	0.9632 (0.8833, 1.0503)
*2DS2*	543 (21.56)	3880 (21.43)	0.96	1.0077 (0.9105, 1.1152)
*2DS3*	444 (17.63)	3340 (18.44)	0.8236	0.9461 (0.8483, 1.0552)
*2DS4*	2393 (95.00)	17,262 (95.33)	0.8236	0.9308 (0.7682, 1.1278)
*2DS5*	654 (25.96)	4683 (25.86)	0.96	1.0053 (0.9141, 1.1056)
*3DS1*	892 (35.41)	6528 (36.05)	0.8388	0.9725 (0.8914, 1.061)
*2DP1*	2492 (98.93)	17,943 (99.09)	0.8236	0.8487 (0.5638, 1.2776)
*3DP1*	2509 (99.60)	18,084 (99.87)	0.044	0.3330 (0.1591, 0.6972)

OR: odds ratio, which describes the odds of cases being KIR genes carriers to the odds of controls being KIR genes carriers.

### The association between KIR genes and hematopoietic-related diseases

**Figure 1:**
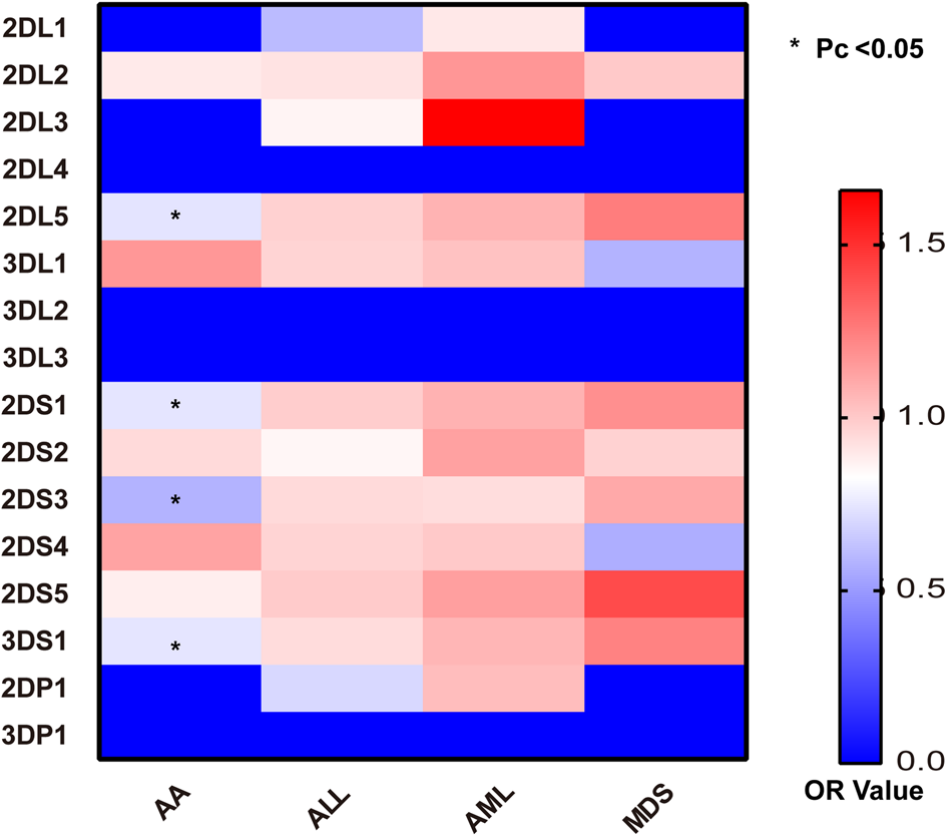
Heat map of KIR gene and various diseases. AA: aplastic anemia; ALL: acute lymphoblastic leukemia; AML: acute myelocytic leukemia; MDS: myelodysplastic syndromes. *Pc < 0.05, *P* values for multiple comparisons were adjusted by Bonferroni correction.

Further analysis of data from various categories of hematology revealed the differences in the KIR genes between patients with hematopoietic-related diseases and controls. Figure 1 shows that the susceptibility of patients with AA and ALL to the KIR genes differed considerably from that of individuals without known pathology (2DL5, 2DS1, 2DS3, and 3DS1 for AA) (Pc < 0.05), and no equivalent involvement appears to be identified in AML and MDS. Other KIR genes including 2DL1-2DL3, 3DL1-3DL3, 2DS2, 2DS4, and two pseudogenes (2DP1 and 3DP1) displayed no significant association with AML and MDS.

**Figure 2:**
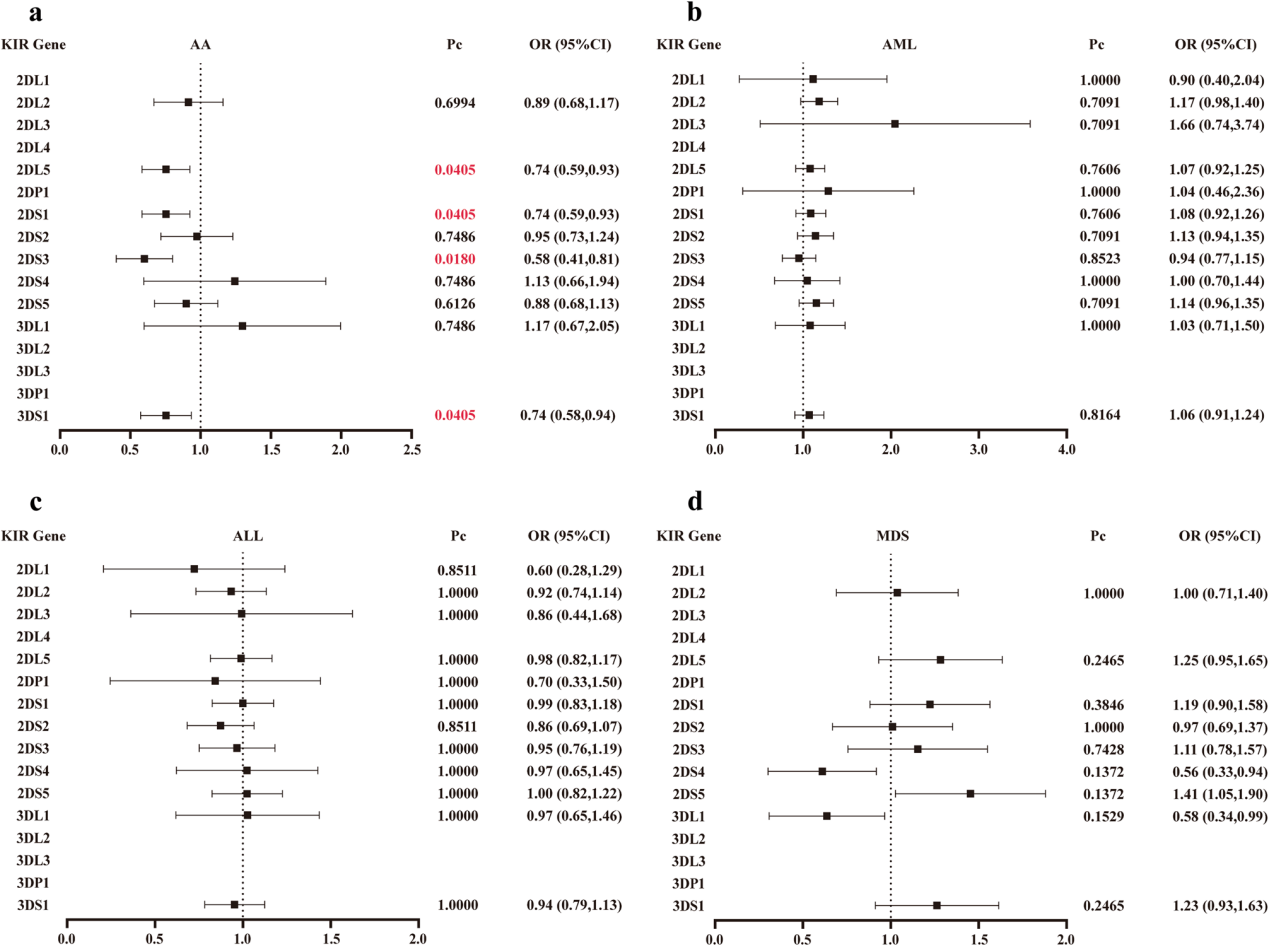
Correlation between KIR gene and disease. (**a**) KIR susceptibility in patients with aplastic anemia; (**b**) KIR susceptibility in patients with acute myelocytic leukemia; (**c**) KIR susceptibility in patients with acute lymphoblastic leukemia; (**d**) KIR susceptibility in patients with myelodysplastic syndromes. CI: confidence interval; OR: odds ratio, which describes the odds of cases being KIR genes carriers to the odds of controls being KIR genes carriers. Pc values for multiple comparisons were corrected by Bonferroni correction.

Furthermore, during the examination of disease-associated genetic polymorphisms, an inhibitory KIR gene and three activating KIR genes were discovered to be protective genes for AA: the former was 2DL5 (OR: 0.74, 95% CI 0.59–0.93; Pc = 0.0405); the latter consisted of 2DS1 (OR: 0.74, 95% CI 0.59–0.93; Pc = 0.0405), 2DS3 (OR: 0.58, 95% CI 0.41–0.81; Pc = 0.0180), and 3DS1 (OR: 0.74, 95% CI 0.58–0.94; Pc = 0.0405) (Fig. 2). On the other hand, it appears that no susceptible KIR genotypes have been associated to AML and MDS. Moreover, we performed further analysis by grouping gender and age according to different disease types, but found that there was no statistically significant difference in the KIR gene frequencies between the different gender groups as well as between the various age groups (Tables S1–S8).

## Discussion

Around the hypothesis that “similar immunogenetic predisposition exists in all or specific subgroups of hematology”, we conducted a retrospective study in a large-scale multi-center Chinese population to analyze the susceptibility of KIR genes in ALL, AML, AA, and MDS. As a result, our study successfully identified the KIR protective genes associated with AA (2DL5, 2DS1, 2DS3, and 3DS1). These findings could contribute reliable evidence for a potential association between KIR genes and hematopoietic-related diseases. This provides more in-depth basic research for clinical treatment.

As a multi-gene family, KIR gene diversity is achieved through differences in gene content and genetic polymorphism. In this study, we found that inhibitory genes (i.e., 2DL1, 2DL3, 3DL2, and 3DL3), activating and inhibiting potential genes (i.e., 2DL4), and pseudogenes (2DP1 and 3DP1) of KIR were common in all patients and controls. On the contrary, the frequency of activating KIR genes (excepting 2DS4), was lower than that of all inhibitory KIR genes except 2DL2 and 2DL5. Consistently, in a study based on the Han population in northern China, the frequencies of KIR3DL3, 3DL2, and 2DL4 reached 100% in all subjects (including 50 leukemia patients and 60 healthy donors)^19^. Strong linkage disequilibrium between KIR genes may contribute to this phenomenon, causing it hard to determine which KIR genes are associated with a specific disease. Consequently, it is extremely necessary to classify different types of diseases to obtain accurate results.

Interestingly, several reports have identified an association between certain KIR genes and hematological malignancies. For instance, Tao et al. used PCR-SSP to analyze KIR genes in 253 AML patients and 273 healthy individuals of Han nationality in southern China. It is indicated that the frequency of KIR2DS4 was significantly higher in AML patients than that in controls^20^. Similarly, Serio et al. found a decreased frequency of the 2DL3 gene in AA patients^21^. Another study suggested that MDS patients had higher frequencies of 2DS5 and lower frequencies of 2DL3 when compared to healthy individuals^22^. Hence, to eliminate the effect of multiple diseases confounding the outcomes, we subdivided the blood disorders in the next analysis into four types: AML, ALL, AA, and MDS, with MDS progressing to AML. This enables precise and comprehensive relationships between genetic variations in KIR gene cluster and risk of hematopoietic related diseases.

In view of the various immunogenetic factors, KIR genes may play an important role in enhancing and/or reducing the immune response^23–26^. Dou et al. investigated KIR2DL1 in AML patients compared with donors (17.6% vs. 75%, Pc = 0.03)^27^. Our inconsistent results with this report require further advanced research to follow up. On the other hand, by comparing the susceptibility to KIR genes in AA and MDS patients with controls, 2DL5, 2DS1, 2DS3, and 3DS1 were found to be protective factors for AA. In this regard, no association was found between the KIR genotype and MDS. Based on this, Serio et al. reported a decreased frequency of the inhibitory KIR2DL3 gene for AA but no difference in the frequency of the KIR genotype for MDS^21^, which is consistent with our findings. Moreover, it has been shown that KIR genotypes and cytokines, as well as cytokine receptor gene SNPs, may play a role in the important susceptibility to excessive and/or reduced immune response^28,29^. For instance, expression of the KIR3DS1 ligand HLA-F is selectively lost on KIR-L (-) primitive hematopoietic stem cells derived from 6pLOH (+) induced pluripotent stem cells in KIR3DS1 (+) patients^30^. And Zeng et al. found an association between toll-like receptor genes in bone marrow CD4+ cells and overexpression of KIR genes in bone marrow CD8+ cells in AA patients^31^. Overall, following the above studies, the relationship between susceptibility genes and the severity and response to AA demands further confirmation.

With several limitations of previous research, our study is the first large-scale multi-center study on KIR in China. The purpose of the study was to explore the frequency distribution and susceptibility of KIR genes among AML, ALL, AA, and MDS patients and individuals without known pathology. Although some of our findings appear inconsistent with previous studies, possible explanations for the discrepancy lie in the differences in cohort size, disease classification, and significant ethnic and regional distribution bias. Besides, despite the diagnostic information of some patients being missing or unclear, a relatively large sample size may attenuate the impact of partial information loss.

## Methods

### Study population

A total of 20,627 peripheral blood samples (11,523 males, 8896 females) from the Chinese Han population were collected in over 237 hospitals mainly concentrated in southeastern China between 8 June 2012 and 17 July 2021. The age range was from 1 month to 73 years (median, 33 years), of which 18,156 were older than 14 years old. There were 2519 patients with hematological-related diseases and 18,108 individuals without known pathology. Among the enrolled patients, there were 539 diagnosed with ALL, 686 with AML, 200 with MDS, 336 with AA, and 758 with other types of diseases (including thalassemia, hemophagocytic lymphohistiocytosis, and multiple myeloma, etc.). The written informed consent was obtained from all individual participants in accordance with the Declaration of Helsinki. The study was approved by the Ethics Committee of Shanghai Tissuebank Medical Laboratory.

### DNA extraction

Genomic DNA was extracted from peripheral blood using the QIARamp Blood Kit (Qiagen, Hilden, Germany). The purified DNA was dissolved in 0.5 mL of DNA hydration solution and stored at -80℃ for future use.

### KIR genotyping

The presence or absence of the KIR genes was determined using the Dynal KIR Genotyping kit (Invitrogen Corp, Carlsbad, CA, USA). Genomic DNA was amplified by polymerase chain reaction with specific sequence primers (PCR-SSP) and mixed with 31 μL (50 ng/μL) of genomic DNA sample and reaction buffer, 2.3 μL Taq DNA polymerase (5 U/μL), and 152 μL of water. PCR amplification began with denaturation at 95 °C for 1 min, followed by 30 cycles of amplification at 94 °C for 20 s, 63 °C for 20 s, and 72 °C for 90 s. The PCR products were analyzed on 2% agarose gel. This method was used to type 14 KIR genes and 2 pseudogenes: 2DL1-2DL5, 2DS1-2DS5, 3DL1-3DL3, 3DS1, 2DP1, and 3DP1. KIR genotype frequencies were estimated by counting the number of positive individuals for a given gene in 2% agarose gel.

### Statistical analysis

All analyses were performed using R version 4.1.0. The chi-square test was applied to compare the variability of each KIR gene in patients and controls. GraphPad Prism 9 was used to compare the frequency distribution of each KIR gene. Prevalence among patients was counted according to diagnostic information and the strength of association was estimated as ORs with 95% CIs. Then pairwise comparisons were adjusted for multiple tests with Bonferroni's correction. An adjusted Pc < 0.05 indicates a statistical significance.

### Ethics approval

This is an observational study. The XYZ Research Ethics Committee of Shanghai Tissuebank Medical Laboratory has confirmed that no ethical approval is required.

### Consent to participate

Informed consent was obtained from all individual participants included in the study.

## Conclusions

Our study has explored the susceptibility of KIR genotypes associated with hematopoietic-related diseases in the Chinese Han population. We need to discover KIR genes, ligands, and functions, as well as understand the role of KIR genes in hematopoietic-related disease pathways. It can provide further insight into donor selection for Chinese transplant patients in clinical practice.

## Supplementary Information

Supplementary Information.

## Data Availability

The datasets generated and/or analyzed during the current study are not publicly available due to privacy, the data can not be published publicly but are available from the corresponding author on reasonable request.
