# Supplementary material for: Exploration of KIR genes and hematological-related diseases in Chinese Han population
Source: Sci Rep. 2023 Jun 16;13:9773. doi: 10.1038/s41598-023-36882-y (PMC10276034; doi:10.1038/s41598-023-36882-y)
Supplement: Supplementary file 1 — Supplementary Information. [file 41598_2023_36882_MOESM1_ESM.docx]

**Table S1.** **KIR gene frequencies in AA patients and controls by sex group**

| **Gene** | **Male** | | **Pc** | **OR (95%CI)** | **Female** | | **Pc** | **OR (95%CI)** |
| --- | --- | --- | --- | --- | --- | --- | --- | --- |
|  | **Patients**  **N(%)** | **Controls**  **N(%)** |  |  | **Patients**  **N(%)** | **Controls**  **N(%)** |  |  |
| ***2DL1*** | 178 (99.44) | 9945 (99.27) | - | - | 157 (100.00) | 7818 (99.14) | - | - |
| ***2DL2*** | 38 (21.23) | 2183 (21.79) | 1 | 0.9673  (0.6739, 1.3884) | 28 (17.83) | 1653 (20.96) | 0.5227 | 0.8185  (0.5420, 1.2361) |
| ***2DL3*** | 177 (98.88) | 9891 (98.73) | - | - | 157 (100.00) | 7754 (98.33) | - | - |
| ***2DL4*** | 178 (99.44) | 10004 (99.86) | - | - | 157 (100.00) | 7876 (99.87) | - | - |
| ***2DL5*** | 64 (35.75) | 4123 (41.16) | 0.6822 | 0.7957  (0.5846, 1.0830) | 51 (32.48) | 3216 (40.78) | 0.0945 | 0.6987  (0.4988, 0.9787) |
| ***3DL1*** | 171 (95.53) | 9570 (95.53) | 1 | 1.0006  (0.4893, 2.0460) | 152 (96.82) | 7531 (95.50) | 0.5227 | 1.4330  (0.5843, 3.5147) |
| ***3DL2*** | 178 (99.44) | 10010 (99.92) | - | - | 157 (100.00) | 7880 (99.92) | - | - |
| ***3DL3*** | 178 (99.44) | 10010 (99.92) | - | - | 157 (100.00) | 7879 (99.91) | - | - |
| ***2DS1*** | 59 (32.96) | 3759 (37.52) | 0.6822 | 0.8187  (0.5979, 1.1210) | 45 (28.66) | 2926 (37.10) | 0.0945 | 0.6811  (0.4805, 0.9655) |
| ***2DS2*** | 40 (22.35) | 2186 (21.82) | 1 | 1.031  (0.7230, 1.4702) | 29 (18.47) | 1645 (20.86) | 0.5227 | 0.8596  (0.5723, 1.2910) |
| ***2DS3*** | 23 (12.85) | 1837 (18.34) | 0.6657 | 0.6566  (0.4226, 1.0202) | 16 (10.19) | 1469 (18.63) | 0.0832 | 0.4957  (0.2947, 0.8339) |
| ***2DS4*** | 170 (94.97) | 9552 (95.35) | 1 | 0.9215  (0.4683, 1.8132) | 152 (96.82) | 7515 (95.30) | 0.5227 | 1.5008  (0.6121, 3.6800) |
| ***2DS5*** | 44 (24.58) | 2617 (26.12) | 1 | 0.9217  (0.6540, 1.2990) | 36 (22.93) | 2010 (25.49) | 0.5227 | 0.8698  (0.5975, 1.2662) |
| ***3DS1*** | 58 (32.40) | 3611 (36.05) | 0.8051 | 0.8505  (0.6203, 1.1662) | 42 (26.75) | 2849 (36.13) | 0.0832 | 0.6457  (0.4521, 0.9221) |
| ***2DP1*** | 178 (99.44) | 9936 (99.18) | - | - | 157 (100.00) | 7805 (98.97) | - | - |
| ***3DP1*** | 178 (99.44) | 10005 (99.87) | - | - | 157 (100.00) | 7875 (99.86) | - | - |

AA: aplastic anemia；CI: confidence interval; OR: odds ratio, which describes the odds of cases being KIR genes carriers to the odds of controls being KIR genes carriers.

**Table S2.** **KIR gene frequencies in AML patients and controls by sex group**

| **Gene** | **Male** | | **Pc** | **OR (95%CI)** | **Female** | | **Pc** | **OR (95%CI)** |
| --- | --- | --- | --- | --- | --- | --- | --- | --- |
|  | **Patients**  **N(%)** | **Controls**  **N(%)** |  |  | **Patients**  **N(%)** | **Controls**  **N(%)** |  |  |
| ***2DL1*** | 360 (98.63) | 9945 (99.27) | 0.487 | 0.5285  (0.2123, 1.3158) | 320 (99.69) | 7818 (99.14) | - | - |
| ***2DL2*** | 88 (24.11) | 2183 (21.79) | 0.5269 | 1.1402  (0.8929, 1.4560) | 78 (24.30) | 1653 (20.96) | 0.394 | 1.2104  (0.9326, 1.571) |
| ***2DL3*** | 360 (98.63) | 9891 (98.73) | 1 | 0.9245  (0.3760, 2.2734) | 320 (99.69) | 7754 (98.33) | - | - |
| ***2DL4*** | 365 (100.00) | 10004 (99.86) | - | - | 321 (100.00) | 7876 (99.87) | - | - |
| ***2DL5*** | 163 (44.66) | 4123 (41.16) | 0.487 | 1.1537 (0.9350, 1.4235) | 130 (40.50) | 3216 (40.78) | 0.958 | 0.9884  (0.7874, 1.2407) |
| ***3DL1*** | 342 (93.70) | 9570 (95.53) | 0.487 | 0.6961  (0.4516, 1.0730) | 314 (97.82) | 7531 (95.50) | 0.3081 | 2.1145  (0.9923, 4.5058) |
| ***3DL2*** | 365 (100.00) | 10010 (99.92) | - | - | 321 (100.00) | 7880 (99.92) | - | - |
| ***3DL3*** | 365 (100.00) | 10010 (99.92) | - | - | 321 (100.00) | 7879 (99.91) | - | - |
| ***2DS1*** | 145 (39.73) | 3759 (37.52) | 0.5319 | 1.0974  (0.8864, 1.3586) | 123 (38.32) | 2926 (37.10) | 0.8726 | 1.0530  (0.8370, 1.3248) |
| ***2DS2*** | 86 (23.56) | 2186 (21.82) | 0.5319 | 1.1044  (0.8633, 1.4129) | 76  (23.68) | 1645 (20.86) | 0.394 | 1.1769  (0.9047, 1.5310) |
| ***2DS3*** | 70 (19.18) | 1837 (18.34) | 0.7851 | 1.0568 (0.8104,1.3781) | 50  (15.58) | 1469 (18.63) | 0.394 | 0.8060  (0.5930, 1.0956) |
| ***2DS4*** | 341 (93.42) | 9552 (95.35) | 0.487 | 0.6932  (0.4535, 1.0595) | 313 (97.51) | 7515 (95.30) | 0.3081 | 1.9315  (0.9501, 3.9265) |
| ***2DS5*** | 109 (29.86) | 2617 (26.12) | 0.487 | 1.2041  (0.9581, 1.5133) | 86  (26.79) | 2010 (25.49) | 0.8726 | 1.0698  (0.8314, 1.3766) |
| ***3DS1*** | 142 (38.90) | 3611 (36.05) | 0.4918 | 1.1298  (0.9118, 1.3999) | 115 (35.83) | 2849 (36.13) | 0.958 | 0.9870  (0.7821, 1.2456) |
| ***2DP1*** | 360 (98.63) | 9936 (99.18) | 0.5319 | 0.5942  (0.2394, 1.4747) | 320 (99.69) | 7805 (98.97) | - | - |
| ***3DP1*** | 365 (100.00) | 10005 (99.87) | - | - | 321 (100.00) | 7875 (99.86) | - | - |

AML: acute myelocytic leukemia; CI: confidence interval; OR: odds ratio, which describes the odds of cases being KIR genes carriers to the odds of controls being KIR genes carriers.

**Table S3.** **KIR gene frequencies in ALL patients and controls by sex group**

| **Gene** | **Male** | | **Pc** | **OR (95%CI)** | **Female** | | **Pc** | **OR (95%CI)** |
| --- | --- | --- | --- | --- | --- | --- | --- | --- |
|  | **Patients**  **N(%)** | **Controls**  **N(%)** |  |  | **Patients**  **N(%)** | **Controls**  **N(%)** |  |  |
| ***2DL1*** | 333 (98.52) | 9945 (99.27) | 0.6916 | 0.4889  (0.1963, 1.2178) | 199 (99.00) | 7818 (99.14) | - | - |
| ***2DL2*** | 69 (20.41) | 2183 (21.79) | 0.7971 | 0.9206  (0.7037, 1.2044) | 38  (18.91) | 1653 (20.96) | 0.5555 | 0.8791  (0.6150, 1.2565) |
| ***2DL3*** | 333 (98.52) | 9891 (98.73) | 0.8972 | 0.8551  (0.3476, 2.1038) | 197 (98.01) | 7754 (98.33) | - | - |
| ***2DL4*** | 334 (98.82) | 10004 (99.86) | - | - | 199 (99.00) | 7876 (99.87) | - | - |
| ***2DL5*** | 142 (42.01) | 4123 (41.16) | 0.8972 | 1.0359  (0.8316, 1.2903) | 75  (37.31) | 3216 (40.78) | 0.4337 | 0.8644  (0.6472, 1.1545) |
| ***3DL1*** | 318 (94.08) | 9570 (95.53) | 0.6916 | 0.7443  (0.4691, 1.1810) | 196 (97.51) | 7531 (95.50) | 0.4337 | 1.8478  (0.7558, 4.5177) |
| ***3DL2*** | 336 (99.41) | 10010 (99.92) | - | - | 199 (99.00) | 7880 (99.92) | - | - |
| ***3DL3*** | 336 (99.41) | 10010 (99.92) | - | - | 199 (99.00) | 7879 (99.91) | - | - |
| ***2DS1*** | 132 (39.05) | 3759 (37.52) | 0.7971 | 1.0669  (0.8543, 1.3324) | 67  (33.33) | 2926 (37.10) | 0.4337 | 0.8476  (0.6299, 1.1405) |
| ***2DS2*** | 66 (19.53) | 2186 (21.82) | 0.6916 | 0.8694  (0.6616, 1.1424) | 35  (17.41) | 1645 (20.86) | 0.4337 | 0.7999  (0.5533, 1.1564) |
| ***2DS3*** | 57 (16.86) | 1837 (18.34) | 0.7971 | 0.9034  (0.6765, 1.2063) | 38  (18.91) | 1469 (18.63) | 0.9255 | 1.0184  (0.7122, 1.4562) |
| ***2DS4*** | 317 (93.79) | 9552 (95.35) | 0.6916 | 0.7364  (0.4689, 1.1564) | 196 (97.51) | 7515 (95.30) | 0.4337 | 1.9352  (0.7917, 4.7302) |
| ***2DS5*** | 97 (28.70) | 2617 (26.12) | 0.6916 | 1.1383  (0.8955, 1.4469) | 41  (20.40) | 2010 (25.49) | 0.4337 | 0.7491  (0.5296, 1.0596) |
| ***3DS1*** | 121 (35.80) | 3611 (36.05) | 0.9560 | 0.9894  (0.7892, 1.2404) | 65  (32.34) | 2849 (36.13) | 0.4337 | 0.8450  (0.6266, 1.1396) |
| ***2DP1*** | 334 (98.82) | 9936 (99.18) | - | - | 198 (98.51) | 7805 (98.97) | - | - |
| ***3DP1*** | 335 (99.11) | 10005 (99.87) | - | - | 200 (99.50) | 7875 (99.86) | - | - |

ALL: acute lymphoblastic leukemia; CI: confidence interval; OR: odds ratio, which describes the odds of cases being KIR genes carriers to the odds of controls being KIR genes carriers.

**Table S4.** **KIR gene frequencies in MDS patients and controls by sex group**

| **Gene** | **Male** | | **Pc** | **OR (95%CI)** | **Female** | | **Pc** | **OR (95%CI)** |
| --- | --- | --- | --- | --- | --- | --- | --- | --- |
|  | **Patients**  **N(%)** | **Controls**  **N(%)** |  |  | **Patients**  **N(%)** | **Controls**  **N(%)** |  |  |
| ***2DL1*** | 121 (100.00) | 9945 (99.27) | - | - | 79 (100.00) | 7818 (99.14) | - | - |
| ***2DL2*** | 25 (20.66) | 2183 (21.79) | 0.9293 | 0.9347  (0.6004, 1.4552) | 17  (21.52) | 1653 (20.96) | 1.0000 | 1.0339  (0.6029, 1.773) |
| ***2DL3*** | 119 (98.35) | 9891 (98.73) | - | - | 78  (98.73) | 7754 (98.33) | - | - |
| ***2DL4*** | 120 (99.17) | 10004 (99.86) | - | - | 78  (98.73) | 7876 (99.87) | - | - |
| ***2DL5*** | 57 (47.11) | 4123 (41.16) | 0.4397 | 1.2734  (0.8892, 1.8237) | 36  (45.57) | 3216 (40.78) | 0.6214 | 1.2157  (0.7790, 1.8972) |
| ***3DL1*** | 111 (91.74) | 9570 (95.53) | 0.2384 | 0.5196  (0.2702, 0.9993) | 74  (93.67) | 7531 (95.50) | 0.7357 | 0.6976  (0.2803, 1.7364) |
| ***3DL2*** | 120 (99.17) | 10010 (99.92) | - | - | 78  (98.73) | 7880 (99.92) | - | - |
| ***3DL3*** | 120 (99.17) | 10010 (99.92) | - | - | 78  (98.73) | 7879 (99.91) | - | - |
| ***2DS1*** | 49 (40.50) | 3759 (37.52) | 0.8381 | 1.1332  (0.7865, 1.6328) | 34  (43.04) | 2926 (37.10) | 0.6214 | 1.2808  (0.8185, 2.0041) |
| ***2DS2*** | 25 (20.66) | 2186 (21.82) | 0.9293 | 0.9330  (0.5993, 1.4525) | 16  (20.25) | 1645 (20.86) | 1.0000 | 0.9635  (0.5551, 1.6723) |
| ***2DS3*** | 22 (18.18) | 1837 (18.34) | 1 | 0.9897  (0.6218, 1.5752) | 18  (22.78) | 1469 (18.63) | 0.6214 | 1.2890  (0.7596, 2.1873) |
| ***2DS4*** | 111 (91.74) | 9552 (95.35) | 0.2384 | 0.5415  (0.2816, 1.0412) | 73  (92.41) | 7515 (95.30) | 0.6214 | 0.6006  (0.2596, 1.3897) |
| ***2DS5*** | 40 (33.06) | 2617 (26.12) | 0.2384 | 1.3966  (0.9538, 2.0451) | 26  (32.91) | 2010 (25.49) | 0.6214 | 1.4341  (0.8945, 2.2992) |
| ***3DS1*** | 47 (38.84) | 3611 (36.05) | 0.8381 | 1.1269  (0.7801, 1.6279) | 35  (44.30) | 2849 (36.13) | 0.6214 | 1.4064  (0.9001, 2.1975) |
| ***2DP1*** | 120 (99.17) | 9936 (99.18) | - | - | 78  (98.73) | 7805 (98.97) | - | - |
| ***3DP1*** | 120 (99.17) | 10005 (99.87) | - | - | 78  (98.73) | 7875 (99.86) | - | - |

MDS: myelodysplastic syndromes; CI: confidence interval; OR: odds ratio, which describes the odds of cases being KIR genes carriers to the odds of controls being KIR genes carriers.

**Table S5.** **KIR gene frequencies in AA patients and controls by age group**

| **Gene** | **Age (≤14 years old)** | | **Pc** | **OR (95%CI)** | **Age (>14 years old)** | | **Pc** | **OR (95%CI)** |
| --- | --- | --- | --- | --- | --- | --- | --- | --- |
|  | **Patients**  **N(%)** | **Controls**  **N(%)** |  |  | **Patients**  **N(%)** | **Controls**  **N(%)** |  |  |
| ***2DL1*** | 171 (99.42) | 1296 (99.39) | - | - | 164 (100.00) | 16467 (99.2) | - | - |
| ***2DL2*** | 30 (17.44) | 290 (22.24) | 0.3103 | 0.7387  (0.4878, 1.1186) | 36  (21.95) | 3546 (21.36) | 1 | 1.0354  (0.7140, 1.5014) |
| ***2DL3*** | 170 (98.84) | 1288 (98.77) | - | - | 164 (100.00) | 16357 (98.54) | - | - |
| ***2DL4*** | 171 (99.42) | 1304 (100.00) | - | - | 164 (100.00) | 16576 (99.86) | - | - |
| ***2DL5*** | 54 (31.40) | 489 (37.50) | 0.3103 | 0.7627  (0.5423, 1.0726) | 61  (37.20) | 6850 (41.27) | 0.94 | 0.8430  (0.6133, 1.1588) |
| ***3DL1*** | 166 (96.51) | 1262 (96.78) | 1 | 0.9208  (0.3855, 2.1992) | 157 (95.73) | 15839 (95.42) | 1 | 1.0776  (0.5036, 2.3057) |
| ***3DL2*** | 171 (99.42) | 1304 (100.00) | - | - | 164 (100.00) | 16586 (99.92) | - | - |
| ***3DL3*** | 171 (99.42) | 1304 (100.00) | - | - | 164 (100.00) | 16585 (99.91) | - | - |
| ***2DS1*** | 48 (27.91) | 435 (33.36) | 0.3103 | 0.7733  (0.5436, 1.1001) | 56  (34.15) | 6250 (37.65) | 0.94 | 0.8587  (0.6209, 1.1876) |
| ***2DS2*** | 32 (18.60) | 292 (22.39) | 0.4228 | 0.7922  (0.5281, 1.1884) | 37  (22.56) | 3539 (21.32) | 1 | 1.0752  (0.7441, 1.5536) |
| ***2DS3*** | 21 (12.21) | 251 (19.25) | 0.2429 | 0.5834  (0.3622, 0.9398) | 18  (10.98) | 3055 (18.40) | 0.1619 | 0.5466  (0.3345, 0.8933) |
| ***2DS4*** | 165 (95.93) | 1260 (96.63) | 0.8822 | 0.8231  (0.3647, 1.8575) | 157 (95.73) | 15807 (95.22) | 1 | 1.1252  (0.5260, 2.4072) |
| ***2DS5*** | 37 (21.51) | 274 (21.01) | 1 | 1.0303  (0.6994, 1.5177) | 43  (26.22) | 4353 (26.22) | 1 | 0.9998  (0.7048, 1.4183) |
| ***3DS1*** | 46 (26.74) | 444 (34.05) | 0.2429 | 0.7071  (0.4950, 1.0100) | 54  (32.93) | 6016 (36.24) | 0.94 | 0.8637  (0.6227, 1.1980) |
| ***2DP1*** | 171 (99.42) | 1295 (99.31) | - | - | 164 (100.00) | 16446 (99.07) | - | - |
| ***3DP1*** | 171 (99.42) | 1304 (100.00) | - | - | 164 (100.00) | 16576 (99.86) | - | - |

AA: aplastic anemia；CI: confidence interval; OR: odds ratio, which describes the odds of cases being KIR genes carriers to the odds of controls being KIR genes carriers.

**Table S6.** **KIR gene frequencies in AML patients and controls by age group**

| **Gene** | **Age (≤14 years old)** | | **Pc** | **OR (95%CI)** | **Age (>14 years old)** | | **Pc** | **OR (95%CI)** |
| --- | --- | --- | --- | --- | --- | --- | --- | --- |
|  | **Patients**  **N(%)** | **Controls**  **N(%)** |  |  | **Patients**  **N(%)** | **Controls**  **N(%)** |  |  |
| ***2DL1*** | 192 (98.97) | 1296 (99.39) | - | - | 488 (99.19) | 16467 (99.20) | - | - |
| ***2DL2*** | 49 (25.26) | 290 (22.24) | 0.8664 | 1.1816  (0.8333, 1.6754) | 117 (23.78) | 3546 (21.36) | 0.3457 | 1.1486  (0.9302, 1.4182) |
| ***2DL3*** | 192 (98.97) | 1288 (98.77) | - | - | 488 (99.19) | 16357 (98.54) | - | - |
| ***2DL4*** | 194 (100.00) | 1304 (100.00) | - | - | 492 (100.00) | 16576 (99.86) | - | - |
| ***2DL5*** | 73 (37.63) | 489 (37.50) | 1 | 1.0055  (0.7365, 1.3728) | 220 (44.72) | 6850 (41.27) | 0.3457 | 1.1512  (0.9612, 1.3788) |
| ***3DL1*** | 191 (98.45) | 1262 (96.78) | - | - | 465 (94.51) | 15839 (95.42) | 0.4374 | 0.8275  (0.5576, 1.2280) |
| ***3DL2*** | 194 (100.00) | 1304 (100.00) | - | - | 492 (100.00) | 16586 (99.92) | - | - |
| ***3DL3*** | 194 (100.00) | 1304 (100.00) | - | - | 492 (100.00) | 16585 (99.91) | - | - |
| ***2DS1*** | 68 (35.05) | 435 (33.36) | 0.9963 | 1.0781  (0.7855, 1.4796) | 200 (40.65) | 6250 (37.65) | 0.3457 | 1.1342  (0.9449, 1.3614) |
| ***2DS2*** | 46 (23.71) | 292 (22.39) | 0.9963 | 1.0772  (0.7549, 1.5371) | 116 (23.58) | 3539 (21.32) | 0.3457 | 1.1386  (0.9216, 1.4067) |
| ***2DS3*** | 29 (14.95) | 251 (19.25) | 0.8664 | 0.7373  (0.4854, 1.1199) | 91  (18.50) | 3055 (18.40) | 1 | 1.0062  (0.7987, 1.2676) |
| ***2DS4*** | 191 (98.45) | 1260 (96.63) | - | - | 463 (94.11) | 15807 (95.22) | 0.3457 | 0.8010  (0.5467, 1.1735) |
| ***2DS5*** | 47 (24.23) | 274 (21.01) | 0.8664 | 1.2019  (0.8432, 1.7131) | 148 (30.08) | 4353 (26.22) | 0.3457 | 1.2104  (0.9952, 1.4721) |
| ***3DS1*** | 65 (33.51) | 444 (34.05) | 1 | 0.9760  (0.7092, 1.3432) | 192 (39.02) | 6016 (36.24) | 0.3457 | 1.1260  (0.9369, 1.3533) |
| ***2DP1*** | 192 (98.97) | 1295 (99.31) | - | - | 488 (99.19) | 16446 (99.07) | - | - |
| ***3DP1*** | 194 (100.00) | 1304 (100.00) | - | - | 492 (100.00) | 16576 (99.86) | - | - |

AML: acute myelocytic leukemia; CI: confidence interval; OR: odds ratio, which describes the odds of cases being KIR genes carriers to the odds of controls being KIR genes carriers.

**Table S7.** **KIR gene frequencies in ALL patients and controls by age group**

| **Gene** | **Age (≤14 years old)** | | **Pc** | **OR (95%CI)** | **Age (>14 years old)** | | **Pc** | **OR (95%CI)** |
| --- | --- | --- | --- | --- | --- | --- | --- | --- |
|  | **Patients**  **N(%)** | **Controls**  **N(%)** |  |  | **Patients**  **N(%)** | **Controls**  **N(%)** |  |  |
| ***2DL1*** | 173 (97.74) | 1296 (99.39) | - | - | 359 (99.17) | 16467 (99.20) | - | - |
| ***2DL2*** | 38 (21.47) | 290 (22.24) | 1 | 0.9559  (0.6525, 1.4003) | 69 (19.06) | 3546 (21.36) | 1 | 0.8669  (0.6652, 1.1298) |
| ***2DL3*** | 173 (97.74) | 1288 (98.77) | - | - | 357 (98.62) | 16357 (98.54) | 1 | 1.0607  (0.4348, 2.5873) |
| ***2DL4*** | 174 (98.31) | 1304 (100.00) | - | - | 359 (99.17) | 16576 (99.86) | - | - |
| ***2DL5*** | 72 (40.68) | 489 (37.50) | 0.7988 | 1.1429  (0.8298, 1.5742) | 145 (40.06) | 6850 (41.27) | 1 | 0.9511  (0.7690, 1.1763) |
| ***3DL1*** | 169 (95.48) | 1262 (96.78) | 0.7988 | 0.7031  (0.3246, 1.5230) | 345 (95.30) | 15839 (95.42) | 1 | 0.9751  (0.5960, 1.5954) |
| ***3DL2*** | 175 (98.87) | 1304 (100.00) | - | - | 360 (99.45) | 16586 (99.92) | - | - |
| ***3DL3*** | 175 (98.87) | 1304 (100.00) | - | - | 360 (99.45) | 16585 (99.91) | - | - |
| ***2DS1*** | 65 (36.72) | 435 (33.36) | 0.7988 | 1.1594  (0.8364, 1.6072) | 134 (37.02) | 6250 (37.65) | 1 | 0.9733  (0.7845, 1.2075) |
| ***2DS2*** | 37 (20.90) | 292 (22.39) | 1 | 0.9159  (0.6232, 1.3460) | 64 (17.68) | 3539 (21.32) | 0.9395 | 0.7926  (0.6035, 1.0410) |
| ***2DS3*** | 33 (18.64) | 251 (19.25) | 1 | 0.9614  (0.6428, 1.4379) | 62 (17.13) | 3055 (18.40) | 1 | 0.9163  (0.6951, 1.2078) |
| ***2DS4*** | 168 (94.92) | 1260 (96.63) | 0.7988 | 0.6519  (0.3126, 1.3594) | 345 (95.30) | 15807 (95.22) | 1 | 1.0181  (0.6224, 1.6654) |
| ***2DS5*** | 45 (25.42) | 274 (21.01) | 0.7988 | 1.2815  (0.8908, 1.8435) | 93 (25.69) | 4353 (26.22) | 1 | 0.9727  (0.7665, 1.2344) |
| ***3DS1*** | 60 (33.90) | 444 (34.05) | 1 | 0.9933  (0.7129, 1.3839) | 126 (34.81) | 6016 (36.24) | 1 | 0.9393  (0.7549, 1.1688) |
| ***2DP1*** | 174 (98.31) | 1295 (99.31) | - | - | 358 (98.90) | 16446 (99.07) | - | - |
| ***3DP1*** | 176 (99.44) | 1304 (100.00) | - | - | 359 (99.17) | 16576 (99.86) | - | - |

ALL: acute lymphoblastic leukemia; CI: confidence interval; OR: odds ratio, which describes the odds of cases being KIR genes carriers to the odds of controls being KIR genes carriers.

**Table S8.** **KIR gene frequencies in MDS patients and controls by age group**

| **Gene** | **Age (≤14 years old)** | | **Pc** | **OR (95%CI)** | **Age (>14 years old)** | | **Pc** | **OR (95%CI)** |
| --- | --- | --- | --- | --- | --- | --- | --- | --- |
|  | **Patients**  **N(%)** | **Controls**  **N(%)** |  |  | **Patients**  **N(%)** | **Controls**  **N(%)** |  |  |
| ***2DL1*** | 35 (100.00) | 1296 (99.39) | - | - | 165  (100) | 16467 (99.20) | - | - |
| ***2DL2*** | 4  (11.43) | 290 (22.24) | - | - | 38  (23.03) | 3546 (21.36) | 0.7033 | 1.1015  (0.7652, 1.5856) |
| ***2DL3*** | 34 (97.14) | 1288 (98.77) | - | - | 163 (98.79) | 16357 (98.54) | - | - |
| ***2DL4*** | 34 (97.14) | 1304 (100.00) | - | - | 164 (99.39) | 16576 (99.86) | - | - |
| ***2DL5*** | 14 (40.00) | 489 (37.50) | 0.8511 | 1.1111  (0.5598, 2.2053) | 79  (47.88) | 6850 (41.27) | 0.2357 | 1.3075  (0.9619, 1.7773) |
| ***3DL1*** | 32 (91.43) | 1262 (96.78) | - | - | 153 (92.73) | 15839 (95.42) | 0.2357 | 0.6126  (0.3389, 1.1074) |
| ***3DL2*** | 34 (97.14) | 1304 (100.00) | - | - | 164 (99.39) | 16586 (99.92) | - | - |
| ***3DL3*** | 34 (97.14) | 1304 (100.00) | - | - | 164 (99.39) | 16585 (99.91) | - | - |
| ***2DS1*** | 14 (40.00) | 435 (33.36) | 0.8511 | 1.3318  (0.6707, 2.6446) | 69  (41.82) | 6250 (37.65) | 0.4340 | 1.1903  (0.8722, 1.6244) |
| ***2DS2*** | 4  (11.43) | 292 (22.39) | - | - | 37  (22.42) | 3539 (21.32) | 0.7786 | 1.0668  (0.7386, 1.5409) |
| ***2DS3*** | 5  (14.29) | 251 (19.25) | 0.8511 | 0.6992  (0.2686, 1.8201) | 35  (21.21) | 3055 (18.40) | 0.4536 | 1.1937  (0.8202, 1.7373) |
| ***2DS4*** | 32 (91.43) | 1260 (96.63) | - | - | 152 (92.12) | 15807 (95.22) | 0.2357 | 0.5866  (0.3315, 1.0381) |
| ***2DS5*** | 10 (28.57) | 274 (21.01) | 0.8511 | 1.5036  (0.7135, 3.1684) | 56  (33.94) | 4353 (26.22) | 0.1844 | 1.4454  (1.0453, 1.9987) |
| ***3DS1*** | 13 (37.14) | 444 (34.05) | 0.8511 | 1.1446  (0.5712, 2.2938) | 69  (41.82) | 6016 (36.24) | 0.2357 | 1.2645  (0.9266, 1.7257) |
| ***2DP1*** | 34 (97.14) | 1295 (99.31) | - | - | 164 (99.39) | 16446 (99.07) | - | - |
| ***3DP1*** | 34 (97.14) | 1304 (100.00) | - | - | 164 (99.39) | 16576 (99.86) | - | - |

MDS: myelodysplastic syndromes; CI: confidence interval; OR: odds ratio, which describes the odds of cases being KIR genes carriers to the odds of controls being KIR genes carriers.
